# Endotracheal Tube Fixation in Patients With Facial Burns, What Are the Options?

**Published:** 2018-09-28

**Authors:** Patrick Assi, Luis Quiroga, Kevin Gerold, Julie Caffrey

**Affiliations:** Johns Hopkins Burn Center, Johns Hopkins University School of Medicine, Baltimore, MD

**Keywords:** endotracheal tube fixation, facial burns, endotracheal tube stabilization, securing ET tube, intermaxillary fixation screw

## DESCRIPTION

A 52-year-old alcoholic woman sustained a 40% total body surface area full-thickness flame burn to her torso, face, neck, and scalp, as well as an inhalational injury. We describe a valid and safe technique to be used for endotracheal (ET) tube fixation using intermaxillary fixation (IMF) in an edentulous patient with facial burns.

## QUESTIONS

1. What are the challenges in ET tube fixation in patients with facial burns?
2. What factors make this described technique an easy and safe method for ET tube fixation in edentulous patients with facial burns?
3. What are the available options described in the literature for ET tube fixation in facial burns?
4. What are the available options in patients with good dentition?

## DISCUSSION

Intubated patients with severe head and neck burns are challenging in every aspect, including securing the ET tube in place since dislodgement is a life-threatening complication in the context of inhalational injury. Aggressive wound care to the face and delayed staged surgical interventions for excision and grafting are the standard of care for head and neck deep burns. Securing the ET tube in the regular fashion using cloth tapes or tube holders is not ideal. The straps and tape will only increase the level of injury due to increasing friction and will disrupt the skin grafts. A major factor impacting our choice of fixation method is whether the patient is edentulous or not. We believe that the method of choice for ET tube fixation should be safe, easy to perform, readily available, and reproducible.

A 2.0-mm DePuy Synthes IMF screw of 8-mm length was used in our case. It is a fenestrated stainless steel screw with 2 cross-holes. A screwdriver was used to insert the screw in the alveolar process of the maxilla. A 0.4-mm stainless steel wire was used and was guided through the cross-holes to fixate the ET tube. For edentulous patients, fixation of the ET tube using IMF screws has been reported for patients with facial burns or in maxillofacial surgery. Multiple insertion techniques are described.^1^^-^3 The use of the IMF screw is associated with good oral hygiene, and no complications were reported in this context. Possible complications that can be predicted are hardware failure, screw site infection, screw loosening, soft tissue irritation, and accidental screw insertion into the maxillary sinus.^1^^,^^2^ It is a fast, minimally invasive method that can be applied safely, and the screws can be removed easily when tracheostomy becomes indicated or extubation is in order. The main contraindications for the IMF screw are any comminuted, unstable, or displaced maxillary or mandibular fractures.

An external cranial fixation device was used in this population of patients back in 1978 by Hansen. This method is invasive, and wound care becomes harder compared with our method.^4^ Other methods previously described include the use of a nasal bridle to secure an ET tube in a child with facial blistering. This method is readily performed, although it might be avoided in specific cases such as ours where minor/major alar cartilages are completely burned, although these are not necessarily considered contraindications for the use of a nasal bridle. Circummandibular, transpalatal, and even transmaxillary wires can also be used for ET fixation at the expense of soft tissue damage and the increased risk of infection with associated poor oral hygiene.^5^

For patients with good dentition, interdental wire fixation was reported as a valid method where a stainless steel wire was inserted in the interdental spaces and anchoring around 1 or multiple teeth bases. This is the standard method used in our center in patients with good dentition. This method has its challenges and is associated with difficulty with appropriate hygiene and gingival injury. It can cause damage to the base of the teeth and loosen them, especially in the case of poor dentition. Multiple other methods are available such as orthodontic brackets, arch bar, and retention prosthesis. All these methods require specialized dental skills.^2^^,^^6^

The method of choice is dictated by each individual case. The presence of good dentition offers a wider selection of methods that can be used. In edentulous patients, ET tube fixation using IMF screw is a simple and safe method. The risks of this method are low and the advantages are many. This method should be considered in edentulous patients with severe facial burns.

## Figures and Tables

**Figure 1:**
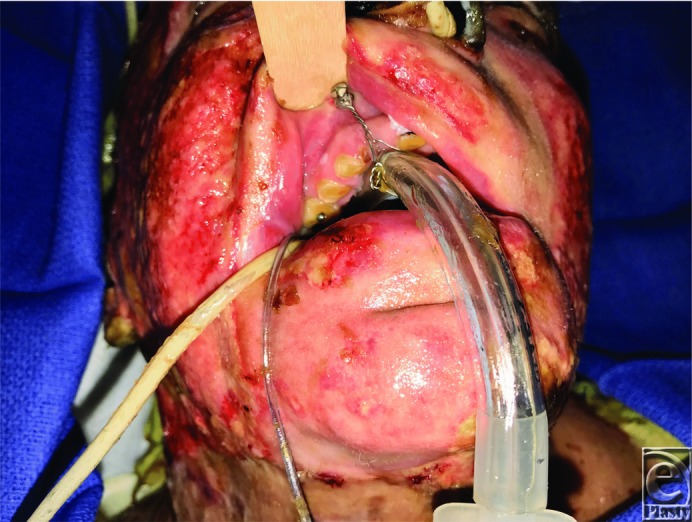
Endotracheal tube fixation using intermaxillary fixation screw in a patient with severe facial and cervical burn.
